# Primary squamous cell carcinoma of the pancreas: A case report

**DOI:** 10.1016/j.ijscr.2025.110962

**Published:** 2025-01-25

**Authors:** Wei Du, Rongyu Shi, Chunlong Shao, Ning Zhang

**Affiliations:** Department of Hepatopancreatobiliary Surgery, First People's Hospital of Jiashan County, Jiaxing, Zhejiang Province, China

**Keywords:** Squamous cell carcinoma, Pancreas, Surgical resection, Case report, Diagnosis

## Abstract

**Introduction:**

Primary squamous cell carcinoma (SCC) is a rare type of pancreatic cancer with an extremely low incidence rate and a prognosis that is poorer than that of pancreatic ductal adenocarcinoma.

**Presentation of case:**

We report a case of pure pancreatic SCC in an 80-year-old man. Based on the examination before surgical resection, we did not detect any SCC lesions that might have metastasized to the pancreas. Partial gastrectomy with caudal pancreatectomy and splenectomy were performed to remove the tumor. Histopathological examination of the resected specimen confirmed the diagnosis of poorly differentiated SCC of the pancreas with stomach invasion and regional lymph node metastasis. The patient was administered adjuvant chemotherapy.

**Discussion:**

SCC poses a diagnostic challenge, requiring histopathology for definitive diagnosis. As an aggressive disease with poor response to chemotherapy and radiotherapy, the prognosis for primary SCC is poor.

**Conclusion:**

Surgical resection and ultrasound-guided fine needle aspiration (EUS-FNA) is helpful for obtaining samples for histopathology and cytology, thus improving diagnostic accuracy.

## Highlights

• Squamous cell carcinoma (SCC) of the pancreas is a controversial entity of uncertain origin, as the pancreas is entirely devoid of squamous cells
• Making a diagnosis based on imaging examinations is highly challenging; It is necessary to use ultrasound-guided fine needle aspiration (EUS-FNA) and surgical resection are required to diagnose pancreatic SCC.
• At present, there is no treatment guidelines have been established for SCC. Genetic test results indicated that trastuzumab combined with chemotherapy was effective in our case report.

## 1 Introduction

Pancreatic cancer is an extremely malignant tumor of the digestive system, characterized by late detection, early metastasis, rapid progression, and poor prognosis. At the time of diagnosis, 30 %–35 % of patients have a locally advanced stage of the tumor, 50 %–55 % have already developed distant metastases, and <20 % are eligible for surgery [1]. Pancreatic cancer ranks tenth in incidence and sixth in mortality in China, with an increasing trend in mortality rates [2]. Primary squamous cell carcinoma (SCC) is a rare type of pancreatic cancer with an extremely low incidence rate and a poor prognosis. Currently, no treatment guidelines have been established for this type of cancer; surgical resection is the primary treatment.

## 2 Case report

**Fig. 1:**
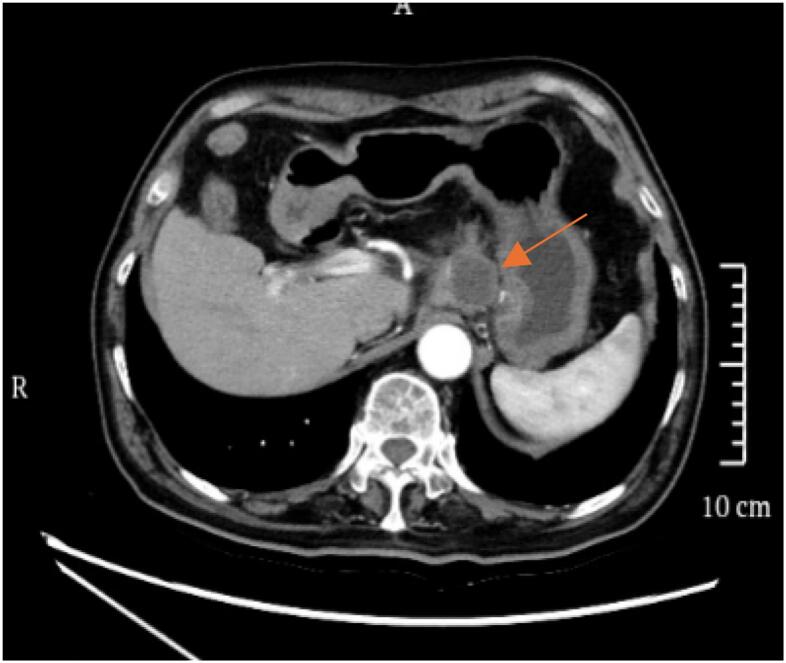
Computed tomography image showing a retroperitoneal mass near the lesser curvature of the stomach, measuring 32 mm × 31 mm.

**Fig. 2:**
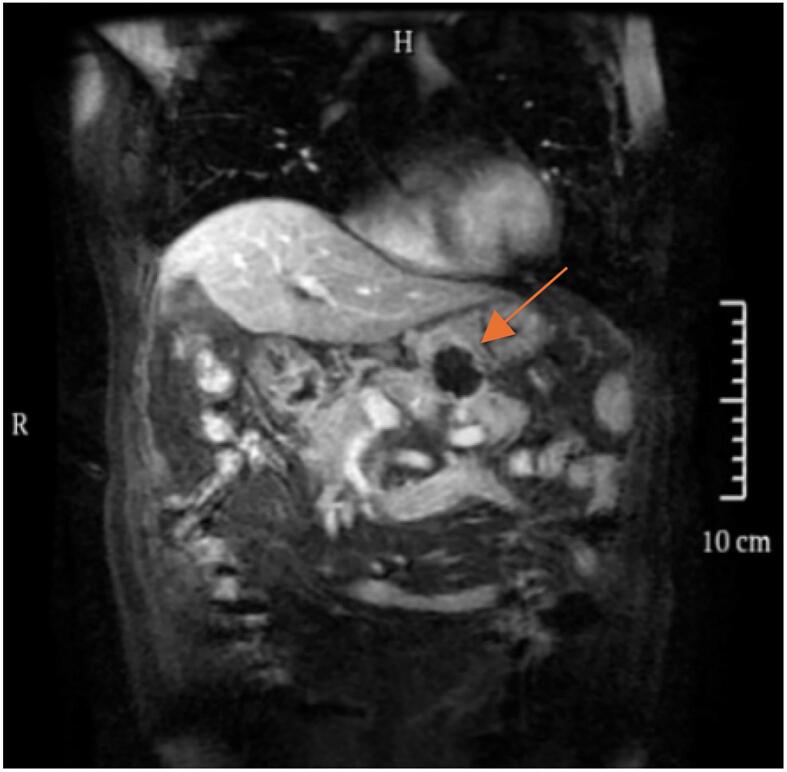
T2-weighted magnetic resonance imaging showing a space-occupying lesion measuring approximately 46 mm × 34 mm in the posterior peritoneum between the pancreas and the lesser curvature of the stomach.

**Fig. 3:**
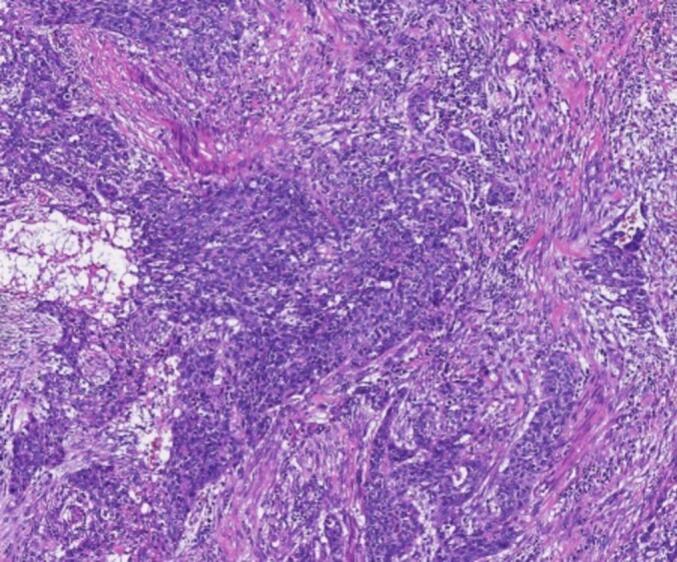
Histopathological findings indicating pure, poorly-differentiated squamous cell carcinoma, with abundant cytoplasmic keratin pearls and intercellular bridges. (Hematoxylin and eosin staining, original magnification ×100).

**Fig. 4:**
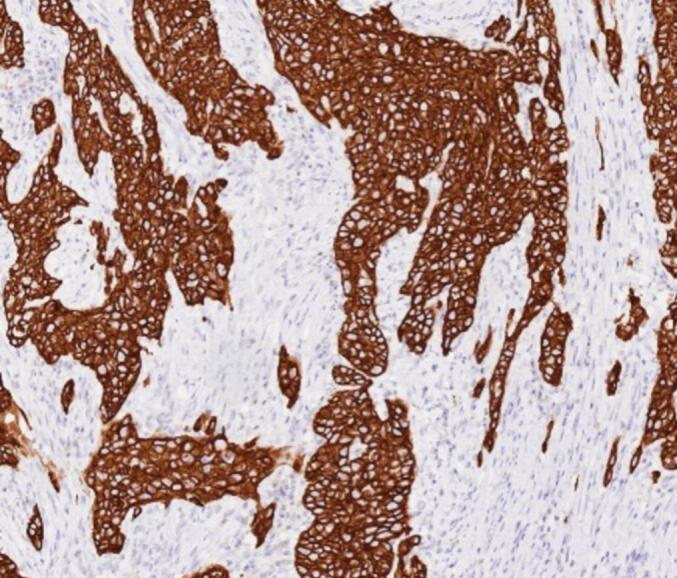
Tumor cells testing positive for CK5/6 expression (CK5/6 immunohistochemistry, ×100).

**Fig. 5:**
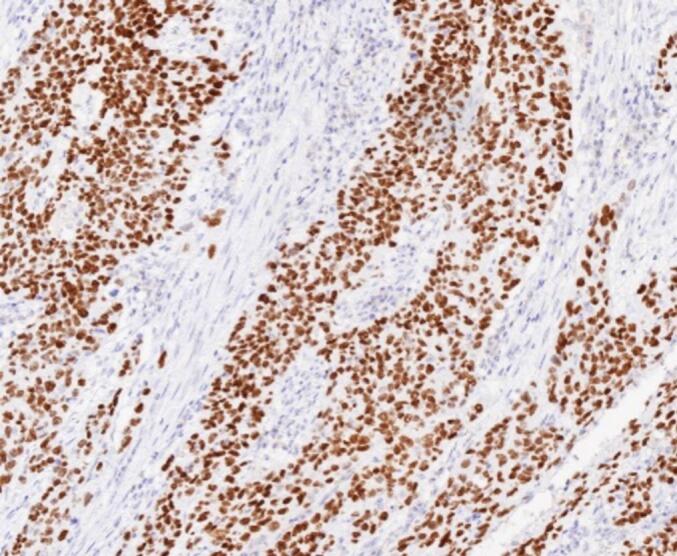
Tumor cells testing positive for P40 expression (p40 immunohistochemistry, ×100).

**Fig. 6:**
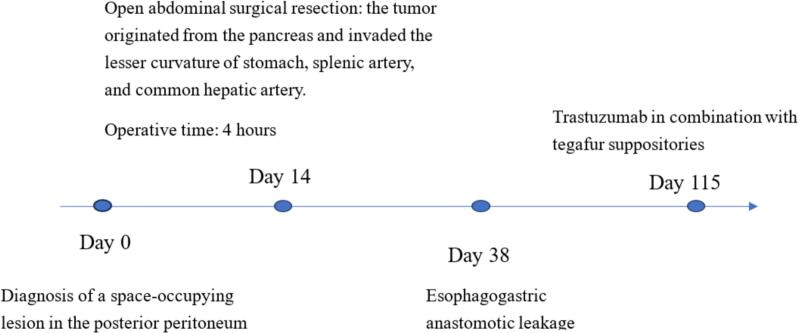
Timeline of patient's course of treatment.

An 80-year-old male patient was hospitalized after a physical examination performed 3 days prior revealed a retroperitoneal mass between the stomach and pancreas without abdominal pain, distension, or fatigue. The patient had a normal appetite and bowel movements, and denied recent weight loss. Physical examination revealed no signs of yellowing of the skin or eyes or superficial lymph node swelling; the abdomen was soft, with no tenderness or rebound pain. The liver and spleen were not enlarged, and no abdominal mass was palpable. The patient had no personal or family history of tumors. Laboratory tests showed the following results: total bilirubin, 17.4 μmol/L (normal, ≤23.0 μmol/L); alanine aminotransferase, 5 U/L (normal, 9–50 U/L); aspartate aminotransferase, 15 U/L (normal, 15–40 U/L), and carbohydrate antigen 19–9 (CA199), 12.1 U/mL (normal, 0–37.0 U/mL). Enhanced computed tomography (CT) of the chest and abdomen revealed a retroperitoneal mass near the lesser curvature of the stomach, with indistinct boundaries between the stomach and pancreas and uneven enhancement patterns (Fig. 1). T2-weighted magnetic resonance imaging (MRI) showed a 46 mm × 34 mm space-occupying lesion in the posterior peritoneum, between the pancreas and the lesser curvature of the stomach. An enhanced scan revealed enhancement at the solid site (Fig. 2). Gastric endoscopy showed no abnormal findings in the upper gastrointestinal tract. Histopathological assessment revealed poorly differentiated pancreatic carcinoma with stomach invasion and regional lymph node metastasis, consistent with SCC (tumor size, approximately 5.5 cm × 4.0 cm × 4.0 cm; Fig. 3). Immunohistochemical analysis revealed that the malignant cells expressed CK5/6 (Fig. 4), p40 (Fig. 5), CK19, HER2 (2+), Ki-67 (+, 80 %), and Brg-1. Tests for CK7, Syn, S100P, INSM1, MUC5AC, and MUC1 showed negative results. Genetic test results indicated the amplification of the tyrosine kinase receptor 2 (*ERBB2*) gene, prompting treatment with trastuzumab in combination with tegafur suppositories (Fig. 6).

This work has been reported in line with the SCARE criteria [3].

## 3 Discussion

Adenocarcinoma is the most common pathological type of pancreatic cancer. In contrast, primary SCC of the pancreas, first reported in 1949, is rare [4] and accounts for 0.5 %–1 % of all pancreatic malignancies [5]. As an aggressive disease with poor response to chemotherapy and radiotherapy, the prognosis for primary SCC is poor [6,7]. In 2016, Makarova-Rusher et al. examined 214 patients with primary pancreatic SCC using the Surveillance, Epidemiology, and End Results Registry (SEER) database and found that the overall survival rate was 14 % at 1 year and 7 % at 2 years. Patients who underwent surgical resection had a 1-year survival rate of 45.3 %, 2-year survival rate of 35.2 %, and median survival rate of 9 months. Patients who received palliative care had a 1-year survival rate of 10.3 %, 2-year survival rate of 3.7 %, and median survival rate of 3 months [8]. In 2019, Tella et al. examined 515 cases of primary pancreatic SCC using the SEER database. They found that for stage I/II disease, regardless of chemotherapy or radiotherapy, the prognosis of the surgical group was better than that of the non-surgical group; the median survival rate of the surgical group was 24 months. In patients with stage III disease, the survival rates of the surgical group and other treatment groups were comparable. Patients with stage IV unresectable disease receiving chemoradiotherapy had higher survival rates [9]. Other studies have confirmed that surgery remains the most effective treatment for patients with resectable disease, prolonging the overall survival time and serving as the only curative treatment [10].

The diagnosis of pancreatic SCC is a matter of exclusion and can only be made in the absence of other primary sites. Imaging-based diagnosis is challenging; therefore, ultrasound-guided fine needle aspiration (EUS-FNA) and surgical resection are required to diagnose pancreatic SCC [11]. Metastatic SCC of the pancreas has been reported previously [12]. Considering that normal pancreatic tissue contains no squamous cells, the mechanism of SCC remains unknown. Currently, four main viewpoints have been proposed. The first proposes that chronic inflammatory stimulation causes squamous metaplasia of the pancreatic ductal epithelium, which can become malignant. A second hypothesis is that it originates from biopotential primitive cells that are capable of differentiating into either glandular adenocarcinoma or SCC. A third viewpoint suggests that preexisting adenocarcinoma with squamous metaplasia transforms into SCC. The fourth proposes that SCC originates from mixed adenosquamous carcinoma in which the glandular components are not visible [[13], [14], [15], [16], [17]].

Immune checkpoint inhibitors have shown great promise in a variety of SCC cancers. Yang et al. opted to treat primary advanced SCC with PD-1 and gemcitabine, achieving partial remission of the neoplasm in the patient. Grant et al. reported complete pathologic response after treatment with gemcitabine, cisplatin, and nab-paclitaxel in a patient with pancreatic cancer with squamous cell differentiation [5,18]. Here, we used trastuzumab to treat SCC, and the patient continues to lead a high-quality life with no symptoms for >4 months. The patient is still being followed up.

## 4 Conclusion

Currently, an effective method for the diagnosis and treatment of pancreatic SCC is lacking. Owing to the strong invasiveness of SCC, most patients experience local progression and metastasis when diagnosed; palliative treatment is ineffective. Surgical treatment can improve patient survival rates. EUS-FNA is a feasible option for inoperable patients; radiotherapy and chemotherapy should be administered based on the histopathology results. PD-1/PD-L1 combined with chemotherapy may be helpful in treatment for of patients with pancreatic SCC. However, more extensive data from a larger research sample are needed to further define the treatment algorithm for pancreatic SCC.

## Author contribution

Wei Du: Conceptualization, Writing – original draft, Writing – review & editing, Data curation.

Rongyu Shi: Supervision, Writing – review & editing, Data curation.

Chunlong Shao: Writing – review & editing.

Ning Zhang: Writing – review & editing.

## Consent

Written informed consent was obtained from the patient for publication of this case report and accompanying images. A copy of the written consent is available for review by the Editor-in-Chief of this journal on request.

## Ethical approval

This study did not require ethical approval as it is a case report (Institution:First People's Hospital of Jiashan county, Jiaxing, Zhejiang Province, China).

## Guarantor

Wei Du.

## Research registration number

Not applicable.

## Funding

This research did not receive any specific grant from funding agencies in the public, commercial, or not-for-profit sectors.

## Conflict of interest statement

The authors declare that there are no conflicts of interest on this case report.
